# Teamwork Competence in Journalism Education: Evidence From TV Organizations’ News Team in Taiwan

**DOI:** 10.3389/fpsyg.2022.864243

**Published:** 2022-06-27

**Authors:** Cheng-Hui Wang, Gloria Hui-Wen Liu, Chia-Dai Yen

**Affiliations:** ^1^Department of Human Resources and Public Relations, Da-Yeh University, Changhua, Taiwan; ^2^International Business School Suzhou (IBSS), Xi’an Jiaotong-Liverpool University, Suzhou, China; ^3^Graduate Institute of Education, National Taiwan Ocean University, Keelung, Taiwan

**Keywords:** journalism education, transactive memory, technology competence, teamwork competence, higher education

## Abstract

The rapid development of digital technologies has transformed the world but can be a double-edged sword. We study the interaction of important variables that affect individual news reporters’ performance in which digital technology is the dominant feature. A multilevel model illustrates how transactive memory and job competence affect individual performance. The empirical study includes data from 19 teams of news reporters and 211 valid survey responses, applying hierarchical linear modeling to analyze the data. The results indicate that transactive memory and technology competence help to improve a reporter’s job performance. More importantly, teamwork competence fully mediates the relationships. Our findings thus suggest that teamwork competence is the core skill. Neither technology competence nor transactive memory alone translates directly into enhanced individual performance.

## Introduction

The development of information and communication technology (ICT) has become a key factor in the economic and social development of countries worldwide, having positive effects on economic growth, productivity, and employment. As digital technologies become the norm in modern life, the manifestations of many human activities are no longer limited to a specific media or a specific time (Mazmanian et al., 2013).

Digitalization opens up the possibility of curricular development to prepare students to deal with new issues (e.g., multimedia news production and tactical media use). However, higher education has tended to respond more slowly to developments in ICT (Hewett, 2016). Especially, the pandemic has massively launched teaching toward the use of ICTs and online education. The massive use of ICTs and educational digitization, and the promotion of SDGs (Sustainable Development Goals) ensure access to quality university education (Rangel-Pérez et al., 2021). Journalism education traditionally includes training in practical skills, on the one hand, and general contextual education and liberal arts training, on the other hand (Deuze, 2004). Forty percent of journalism graduates from US universities feel that there is not enough training in digital technology (Wenger and Owens, 2012). University faculty also reveals that they lack the resources needed to revamp the curriculum to keep up with technological changes (Du and Thornburg, 2011). As more and more journalists are entering the profession *via* higher education, the reform of traditional journalism education becomes pertinent as a way to help develop and sustain professional quality and journalistic identity.

The trend of digital technology has impacted journalism education, and the ranking of university journalism departments has been declining. The importance of journalism education has been challenged due to the rise of the Internet-based self-media. News interviews, editing, and production systems may all be conducted in virtual spaces, and even physical newsrooms may disappear, so journalism education needs to foresee future transformations. Su (2021) believes that Taiwan’s journalism education and industry needs are separated. Journalism schools teach a way, but the industry does another. The news value and business philosophy have created a huge gap between academics and practice. Therefore, students should be more adept at using technology skills in the digital communication environment than before. In addition, the curriculum of journalism education should be project-based to let students be familiar with the overall process of news production in the new digital work environment.

Indeed, digital technology highly influences the professions of journalism (Paulussen, 2012). Digitizing the news production and dissemination process is a common practice for news organizations. They are also increasingly adopting real-time analytics and metrics to monitor and evaluate the performance of individual reporters (Tandoc, 2014). Proficiency with digital technologies becomes essential for reporters (Mirabito et al., 2022).

However, digital technology can be a double-edged sword. News companies are facing the challenges of a steady stream of content enabled by digital technologies (Lewis et al., 2010). There is an increased accusation of inaccurate and irrelevant content by news organizations to maximize their rankings on automated search engines (Carlson, 2018). News organizations must rely on teams to produce professional, balanced reports (Wang et al., 2011) and solve problems swiftly (Deuze, 2005). Team organization is found to facilitate collaboration (Faraj and Xiao, 2006) and exercise clan control on team members (Chua et al., 2012). For example, when reporting an incident, a team of journalists must respond quickly by looking for witnesses to interview, searching for footage of relevant information, triangulating multiple sources of evidence, passing news leads to team members, negotiating and determining relevant topics to report on prioritization, as well as organizing press releases for multimedia platforms (e.g., television and web).

Transactive memory is a team member’s knowledge of the expertise of others (Wegner, 1995; Lewis, 2004; Ren and Argote, 2011). Transactive memory is found to enhance team performance (Ren and Argote, 2011; Bachrach et al., 2019). However, it is unclear how individual reporters on the team utilize transactive memory to improve their performance. The present study aims to look for the areas of competence that need to be emphasized in journalism education to prepare individual students for employment as journalists, given the opportunities and challenges brought by the new communication ecology, with ICT as the dominant feature. This research proposes that teamwork competence and technology competence play key roles in mobilizing transactive memory and digital technology for bettering individual task performance and ensuring the adherence to professional principles of journalism. In the next section, a literature review is presented and a framework is developed outlining how transactive memory, job competencies (i.e., technical competencies and teamwork competencies), and task performance relate to each other.

## Theoretical Framework

Competence is the potential to lead to effective behavioral outcomes (Bassellier et al., 2001). For example, business managers with information technology competencies will be able to create a vision of how information technology contributes to business value and develop strategies to leverage information technology for business growth. The definitions of competence can be grouped into three areas: skill (Willis and Dubin, 1990), personality trait (Hayes, 1979; Spencer and Spencer, 1993), and knowledge (Bassellier et al., 2001). The skills-based perspective assumes that a tight match between predefined tasks and specific skills will lead to superior performance. In the personality-trait approach, competence is defined as the generic knowledge, motive, trait, social role, or skill of a person linked to superior performance on the job (Hayes, 1979). A knowledge-based perspective strikes a middle ground between a skills-based approach and a personality-trait approach. Thus, competence embodies the ability to transfer and apply explicit and tacit knowledge across tasks in complex and changing environments (Brown, 1994). Hence, this research takes a knowledge-based approach, defining competencies as abilities, skills, and tacit knowledge that an individual develops over time.

### Technology Competence

Digital technology is widely used in news organizations. They help increase productivity and better detect and respond to newsworthy events (Wang et al., 2013). Digital technologies have changed the structure of activities in organizations, including the processes of socialization, knowledge sharing, control, and coordination (Treem and Leonardi, 2013). For example, news reporters increasingly use social media for interpersonal communication and interviews with informants. Their competence in harnessing these technologies can thus help improve efficiency and the quality of their work while completing their tasks.

Social media platforms have become important communication tools that allow people to consume as well as provide information content (Ahmed et al., 2019). Over 50% of Americans access news *via* Facebook or Twitter at their convenience as opposed to getting news from television or newspapers at regular times (Gu et al., 2016). News reporters thus also have to harness digital technology to efficiently adapt the news content so that it is suitable to be published on multiple media at the same time (e.g., press, television, internet, and mobile phone). Technology competency is defined as the ability of journalists to understand the capabilities, advantages, limitations, and impacts of digital technologies and use them in the production and dissemination of news content (Bassellier et al., 2001; Deuze, 2004). The digital technologies that journalists need to accomplish their tasks include those that allow them to effectively and efficiently generate news and deliver it to audiences. This research posits that technology competence will allow a news reporter to do a better job because digital technologies offer richer communication channels (e.g., text, audio, and graphics) and an authentic experience (Dennis and Kinney, 1998). Past studies have found that digital technologies can be applied for sourcing stories and informants for interviews, retrieving the history of activities and past contents, delivering text, graphic and audio content, conveying content to the consumer, and receiving comments and feedback (Hirst and Treadwell, 2011; Leonardi et al., 2013). The ability to harness these technologies thus provides the reporter with easy access to various sources of information, enhances their capacity for processing information, and enables better story-telling. Thus, the first hypothesizes is as:

*H1*: The technical competence of journalists is positively related to their task performance.

### Teamwork Competence

Due to increasing competitive pressure and shorter business cycles, teamwork is very common in many organizations (Akgün et al., 2005; Elbanna, 2010). A team is composed of more than two interdependent individuals who are collectively in charge of tasks assigned by the organization (Van der Vegt and Van de Vliert, 2002). An effective team is a collection of individuals, teamwork is more than a collection of individual efforts (Willis and Dubin, 1990). Team members often have different interests and different types of expertise. Conflicts are unavoidable and may include those related to the task at hand (i.e., the content and goals of the job), the process (i.e., how to accomplish the task), and interpersonal issues (i.e., the relationship between team members; Jamshed and Majeed, 2018). However, if the conflict is resolved, it will not be detrimental to the team. Successful resolution of conflicts was found to improve decision-making processes (Darko et al., 2019) and reinforce positive perceptions of team processes by members (Black et al., 2018).

In news reporting, teamwork competency refers to the social and interpersonal knowledge, skills, and abilities (KSA) that journalists need to accomplish team tasks (Stevens and Campion, 1994). KSA for cooperation and problem resolution includes the ability to exchange information, resources, and social support (Tjosvold, 1984) and the ability to integrate different interests or perspectives (Deutsch, 2015). Teamwork competence is particularly salient in fast-paced environments maintaining healthy working relationships and the ability to respond quickly to others with different perspectives, interests, and ideas is critical (Faraj and Xiao, 2006).

Teams in news organizations are often comprised of multiple reporter-videographer pairs, specializing in distinct areas, such as politics, economy, or travel. Take a political news team as an example. Pairs of reporters and videographers on the same team will be stationed in various governmental departments (e.g., Department of National Defense or Department of Foreign Affairs) to cover any political news happening that day related to these departments. Coordination within and between those pairs will include locating needed expertise (internal and external), bringing it to bear on the problem, and solving emerging conflicts to produce news reports quickly which are suited to their specific areas (Faraj and Sproull, 2000). This study considers both the reporter and videographer to be news reporters because of the inseparability of their efforts in producing individual reports. However, it has long been recognized that a news culture that emphasizes individual experts and groupthink over teamwork and knowledge sharing exists (Deuze, 2004). Many studies have reported similar experiences in the newsroom, citing turf wars, and the reporters’ reluctance to share knowledge (Singer, 2004). Perhaps the problem arises because journalism students are usually trained in sequences and because different media (e.g., newspaper and TV) have distinct types of journalism (Deuze, 2006). It is argued that rather than helping students to see the convergence of media, this approach tends to reduce them to individualistic, mono-media journalists, which in turn contributes to the individualistic nature of journalism (Deuze, 2005, 2006).

With the prevalence of digital technology and social media, visibility of communication is likely to promote interaction and open learning among team members (Leonardi et al., 2013). Increased social connections among team members will enhance their commonality. News reporters with teamwork competence thus are sensitive to the common interests of themselves and fellow team members. In fact, this research found that a person’s ability to perceive common interests, downplay opposing interests, and cooperate induces more cooperative behavior and strengthens relationships with others (Van der Vegt and Van de Vliert, 2002; Deutsch, 2015; Liu et al., 2015). People with high teamwork competence can also exercise influence on others by reconstructing their interests, values, or beliefs, leading them to help accomplish assigned tasks (Chua et al., 2012; Liu et al., 2015). In addition, journalists’ ability to resolve conflicts and defuse frictions to prevent infighting (Alper et al., 2000) or compete with others for their own interests (Spreitzer et al., 1999) should conflict arise. Thus, journalism teamwork skills help develop networks and relationships and create social capital that contributes to individual performance. Therefore, this research assumes:

*H2*: Reporters’ teamwork competence is positively related to their task performance.

### Transactive Memory

The concept of transactive memory was first proposed by Wegner (1987), which refers to shared memories between close individuals. This concept was applied to research on teamwork and organizational communication (Lewis, 2004). Transactive memory is defined as “a set of individual memory systems that combine knowledge possessed by a particular member with a shared awareness of who knows what” (Mohammed and Dumville, 2001). It provides “hints” about each member’s unique expertise and expertise. Thus, individual members are able to identify and locate the differentiated knowledge of other members and encourage “free” sharing (Cooke et al., 2000). Previous studies found that group memories are associated with group performance (Ren and Argote, 2011; Bachrach et al., 2019). This is especially true when dealing with complex tasks or when teams lack norms to guide behavior (Akgün et al., 2005). The ecological fallacy comes into play when the results of team performance are used to infer individual performance (Bliese, 2000).

Knowing someone else’s area of expertise is an important coordinating role. News reporting requires an understanding of a variety of potentially useful sources of knowledge and expertise, both internal and external. An individual reporter’s knowledge of individuals across borders can help locate and bring the required expertise to accomplish the task at hand (Faraj and Yan, 2009). Digital technology and social media are argued to help team members absorb external resources and transform the resources into personal ones (Ren and Argote, 2011). As a result, reporters could better utilize each other’s expertise and resources to deliver news more quickly and accurately and even apply them to new tasks. Transactive memory also shapes individual behavior. Individual members can rely on transactive memory to form accurate performance expectations of each other, leading to more efficient allocations based on their capabilities (Kwahk and Park, 2016). This is especially important for teams composed of individuals with different professional roles (Cooke et al., 2000). In such teams, “compatible but disparate knowledge may be critical to task performance” (Bachrach et al., 2019). Some journalists specialize in national defense, some specialize in diplomacy, and still, others specialize in legislation. When a nuclear test event occurs abroad, journalists with relevant expertise want to proactively report on the event, while others play supporting roles and produce other stories related to the event. For example, look back at the global spread of nuclear power over the past few decades. As accurate expectations are formed, individual journalists are more likely to adjust their behavior accordingly and use their resources efficiently to produce news. Therefore, this research assumes as:

*H3*: Transactive memory is positively related to journalists’ task performance.

### The Mediating Effect of Teamwork Competence

It is believed that technical competence helps individual journalists develop a broader perspective on their work. It is generally accepted that the use of enterprise-wide information systems or digital platforms (such as ERP or knowledge management systems) expands employees’ perspectives into cross-functional areas, promotes collaboration, reduces siloed behavior, and fosters the development of teamwork capabilities (Liu et al., 2015).

In news organizations, one of these digitalized platforms would be the broadcast automation system, which specifically is a news editing-broadcasting platform where news reporters can upload, share, select and edit text and video files to meet their specific objectives. The system can help broaden the individual reporters’ attention to relevant events, various types of expertise, and exclusive content owned by individual reporters. In fact, digital technologies create venues for public knowledge sharing and creation, making connections and content visible, and reducing the effort required for coordination (Majchrzak et al., 2013). Reporters with high teamwork competence should be more willing to give up their established way of doing things and work in synergy with colleagues in other parts of the news organization (Deuze, 2005).

On the other hand, although digital technology and social media expedite news production/dissemination processes *via* facilitating collaboration, questions have been raised about the potential of technologies to facilitate deception and cheating without being caught (Light and McGrath, 2010). For example, news reporters could access online chatrooms, perhaps deliberately misrepresenting themselves, to obtain and disseminate information without the informants being informed that it will be published (Whitehouse, 2010).

This research argues that news reporters’ teamwork competence is likely to lead to higher standards of professional integrity and decrease opportunistic, individualistic behaviors in the digitalized workspace. This is because reporters with high teamwork competence are more sensitive to the common interests of the others involved. Furthermore, as journalists engage in more interaction and lasting relationships with other team members, they are likely to be subject to tribal control through peer scrutiny and sanctions (Chua et al., 2012). Thus, individual reporters can not only improve the speed of delivery of tasks but also adhere to the professional principles of journalism. Therefore, this research assumes:

*H4*: Teamwork competence positively mediates the relationship between technology competence and task performance.

Transactive memory provides global knowledge about “who knows what.” However, translating this collective cognition into the realization of distributed intelligence requires a missing link (Wegner, 1987; Ren and Argote, 2011). Teamwork and interdependent interactions among team members provide links that allow complementary information and memory to be stored, retrieved, and processed (Faraj and Sproull, 2000). In other words, teamwork or group interaction is instrumental for mobilizing transactive memory to enhance team performance.

**Figure 1:**
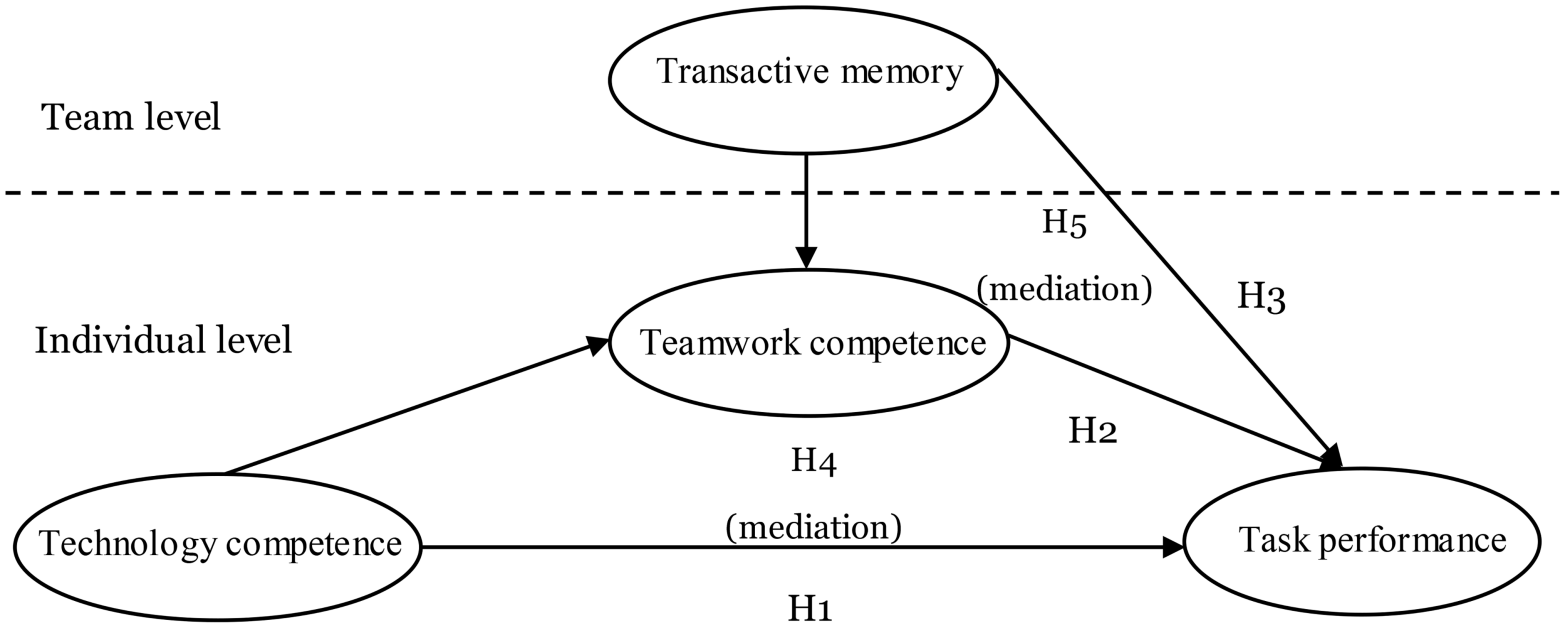
The research framework.

However, individual news reporters are naturally endowed with different capabilities for engaging in cooperative interaction and conflict resolution. Although formal processes (e.g., regular meetings) can be put in place to increase interaction and facilitate utilization of transactive memory, given the non-routine nature of the news generation task, reporters cannot define in advance where and what kind of expertise will be needed (Faraj and Sproull, 2000). Individual reporters need to constantly create and strengthen interactions with others to locate and bring expertise (internal and external) into play. If the interactive relationship persists, reporters are more likely to utilize transactive memory to their own benefit. This is because a long-lasting relationship is more likely to translate into social capital through which transactive memory can be mobilized to one’s favor (Bachrach et al., 2019). For example, reporters are more likely to incorporate multiple perspectives from others into their reporting and treat individual perspectives fairly. Numerous studies have demonstrated that one’s teamwork competence (e.g., trustworthiness or social influence skill) helps with the mobilization of expertise and resources across boundaries (Liu et al., 2011, 2015). Therefore, reporters with higher teamwork competence will be more likely to perform better individually. Consequently, this research proposes the following hypothesis (Figure 1):

H5: Teamwork competence mediates the relationship between transactive memory and task performance.

## Methodology

### Sample

The initial sampling consists of news teams of all TV organizations with nationwide coverage in Taiwan. In total, there were 10 TV organizations geographically divided into three teams, i.e., the north, central, and south. The north teams tend to be further divided and dynamically reorganized because of their proximity to the political capital and major economic hubs. This research collected data from the south and central teams of the 10 TV organizations, i.e., 20 teams in total. The researchers visited each company’s central and south offices to distribute copies of a questionnaire to the leader of each team. The anonymous questionnaire also ensures the privacy of the participants. The filled questionnaires were collected on the same day and directly from individual respondents in blank envelopes. To ensure that each team had enough respondents, this research collected data from teams with at least five reporters and videographers. Finally collected 19 teams with an average team size of 11.1 members. The investigators distributed 300 questionnaires and recovered 211 valid questionnaires (the recovery rate was 70.3%). The study involving human participants was reviewed and approved by National Cheng Kung University Governance Framework for Human Research Ethics (Case Number: 110–560). During the investigation, all the participants in the research understand the purpose of this research and their rights and obligation.

### Measurement

This research obtained most measurements from existing scales and used a 5-point Likert scale. Given that these measures have been translated from English to Mandarin, to avoid semantic ambiguity, this research invited several native English-speaking bilinguals to translate them from Mandarin to English. This research consulted several managers and senior journalists on measures appropriate to our research context.

#### Technology Competence

The technical competency scale was developed through focus group discussions and an extensive review of management and journalism literature (Deuze, 2004; Barebina et al., 2021). Focus groups are moderated by lead authors who have worked in journalism for over 15 years. Focus group participants included three TV news specialists and two HR specialists. This diversity of focus group members represents experts in journalism and human resource management. The discussion sheds light on the common digital technologies that journalists need to produce and distribute news in the age of digital journalism. Three items were developed to measure the technical ability of journalists: (1) I have photography and editing ability, (2) I have multimedia transformation ability, and (3) I have technical ability to handle the immediacy of news production. Cronbach’s Alpha is 0.769.

#### Teamwork Competence

The teamwork competence scale adapted from Youndt and Snell (2004), includes four items: (1) I was able to connect with news sources, (2) I was willing to collaborate with other teammates, (3) I was good at resolving conflicts within the team, and (4) I was willing to share knowledge with teammates. Cronbach’s Alpha is 0.882.

#### Transactive Memory

Transactive memory uses a scale developed by Lewis (2003). Five projects capture this structure: (1) Each team member has expertise in some aspect of news production. (2) I have knowledge of an aspect of news production that other team members do not have. (3) Different team members are responsible for different areas of expertise. (4) The production and delivery of news require the expertise of several different team members. (5) I know which team members have expertise in a particular area. Cronbach’s Alpha is 0.831. The theoretical level of our transactive memory is the team. By citing the team, it focuses on the appropriate level of analysis of the project. For each team, this research aggregated team members’ responses to form a single transactive memory score. To establish whether recognizing the members as a group was appropriate, an investigation of within-group agreement (*r_wg_*) was required (Chan, 1998). The *r_wg_* indices ranged from 0.72 to 0.98 (average transactive memory *r_wg_* = 0.850), indicating acceptable levels of interrater agreement (>0.70; George, 1990). This research also calculated interrater reliability, namely, ICC1 and ICC2 (Biemann et al., 2012). Our results show an ICC1 of 0.236 and an ICC2 of 0.847 (τ00 = 0.069; σ = 0.471). All three indices met the threshold values suggested by prior studies, i.e., 0.70 for *r_wg_*, 0.12 for ICC1, and 0.6 for ICC2 (Cohen, 1988; Bliese, 2000). Based on these results, this research aggregated our data to the team level.

#### Task Performance

Task performance reflects the daily tasks of individual reporters through five items (Wang et al., 2011): (1) I actively plan news coverage. (2) My news reports are timely and accurate. (3) My news reports are fair and objective. (4) My supervisor acknowledges the quality of my scoop. (5) Generally able to handle difficulties and complete news reporting tasks. The reliability test for Cronbach’s Alpha is 0.841.

This study first developed the measure of technology competence, and then assessed its validity by examining its dimensionality, criterion-related validity, and discriminate validity. Finally, conducted the following analysis to demonstrate the validity of technical capabilities in the data. First, examine the dimensionality of this measure by performing a principal component factor analysis using the “Varimax” rotation. A one-factor solution is obtained where all items have a high load with an average load of 80.37%. The single-factor solution explained 70.13% of the variance. Second, examine the validity of the technical competency measure in relation to standards by examining its relationship to other measures that should theoretically be relevant. At the individual level, technical ability is significantly associated with task performance (*r* = 0.429, *p* < 0.01), and transactive memory (*r* = 0.407, *p* < 0.01). An examination of Table 1 demonstrates that the pattern of correlations was consistent with our hypotheses. Finally, used confirmative factor analysis (CFA) to examine the difference between the single factor and multi-factor structure. Table 2 demonstrates the reliability and validity of all our constructs. The single factor of all the items (χ^2^ = 810.55, DF = 119) is significantly different (△χ^2^ = 781.50, *p* < 0.000) from the multi-factor structure (χ^2^ = 298.04, DF = 113). Relevant indicators are in line with the standards of previous studies, which means that the scale of this study has reliability and validity.

**Table 1:** Descriptive statistics and correlations.

	Mean	SD	1	2	3	4	5	6	7
Gender	0.63	0.48	1						
Age	36.31	6.92	0.429^**^	1					
Tenure	10.62	5.79	0.334^**^	0.865^**^	1				
Technology Competence	4.12	0.80	0.344^**^	0.243^**^	0.279^**^	1			
Teamwork Competence	4.22	0.69	0.032	0.119	0.194^**^	0.693^**^	1		
Transactive Memory	4.02	0.69	0.113	0.090	0.172^*^	0.407^**^	0.493^**^	1	
Task Performance	3.90	0.69	−0.061	0.171^*^	0.276^*^	0.429^**^	0.503^**^	0.454^**^	1

* *p < 0.05*;

** *p < 0.01*.

**Table 2:** Reliability and validity of constructs.

	Transactive memory	Technology competence	Teamwork competence	Task performance
Cronbach’s Alpha	0.831	0.769	0.882	0.841
Discriminate validity	*χ*^2^ = 298.04, DF = 113, *χ*^2^ /DF = 2.638, CFI = 0.904, NFI = 0.856, RMSEA = 0.088			

## Analysis and Results

### HLM Null Model

Organizations are hierarchically nested, with individuals nested in groups, groups in departments, and departments in organizations (Kozlowski and Klein, 2000). It is thus very likely that data about individuals (e.g., performance) would be related to group membership. The statistical analysis of these nested relationships requires acknowledgment and correction of the dependency (Bliese, 2000). Hierarchical linear modeling (HLM) provides tests of the relationships that correct for the effects of a group membership. It allows iterative studies of multilevel relationships with individual-level dependent variables (Hofmann et al., 2000). Level 1 analysis estimates parameters that describe the relationship between independent and dependent variables at the individual level. These parameters then become the dependent variables for a level 2 analysis that estimates the effects of group-level variables (e.g., transactive memory). This study explores the relationship between team-level transactive memory and individual-level technology competence, teamwork competence, and task performance. The hierarchical level analysis helps explain the intra and inter team variation.

Before testing the hypotheses using the HLM, it is necessary to estimate a “null model” to ensure there is a dependency between the observations (Kreft and De Leeuw, 1998). A null model or random-effects ANOVA model contains only the dependent variable (i.e., task performance). The model partitions the variance in task performance into individual (level 1) and group (level 2) components and provides an initial estimate for the intra-class correlation (ICC), which is calculated by dividing the level 2 variance by the total variance. ICC is a measure of group homogeneity or the degree of dependence of individuals sharing the same context (Kreft and De Leeuw, 1998).

Results show an ICC1 of 0.333, a variance component (τ_00_) of 0.111, and a level 1 residual (*σ*) of 0.473. An ICC1 of 0.333 means that at most 33.3% of the variance is explained by different group effects. The findings also showed an ICC2 of 0.899. Cohen (1988) suggests that the HLM method is more suitable than the GLM method when the ICC1 is higher than 0.059. Bliese (2000) research shows that an ICC1 greater than 0.138 indicates a large between-group difference, while an ICC2 greater than 0.7 indicates a high intra-group correlation. James (1982) also suggests a threshold of 0.12 for an ICC1 and 0.6 for an ICC2. Our data are thus suitable for analysis using HLM.

### Direct Effects

Table 3 summarizes the results of direct and indirect effects on individual performance. Model 1 and 2 assume that technical competence and teamwork competence will directly affect the task performance of TV news reporters. In Model 1, technical competence showed a significant positive effect on task performance (*γ*_10_ = 0.394, *p* < 0.01). In Model 2, teamwork competence showed similar results, with a significant positive effect on journalists’ task performance (*γ*_10_ = 0.535, *p* < 0.01). Hypothesis 3 proposes group-level effects of transactive memory on individual-level task performance. Using the intercept as the resulting model, Model 3 examined the cross-hierarchical effect of transactive memory on individual task performance. The results showed that transactive memory had a significant positive effect on task performance (γ_01_ = 0.585, *p* < 0.01).

**Table 3:** Regression model of direct and indirect effects on individual task performance.

	Model 1	Model 2	Model 3	Model 4	Model 5
	TECH→TP	TEAM→TP	TM→TP	TECH→TEAM→TP	TM→TEAM→TP
Intercept (γ_00_)	3.891^***^	3.889^***^	3.887^***^	3.886^***^	3.885^***^
TECH (*γ*_10_)	0.394^***^			**0.120**	
TEAM (*γ*_10_) (*γ*_20_)		0.535^***^		0.448^***^	0.516^***^
TM (γ_01_)			0.586^***^		**0.292**
LV1-R	0.373	0.328	0.468	0.300	0.329
Deviance	407.782	387.127	444.180	377.647	384.629

TECH means technology competence; TEAM means team competence; and TM means transactive memory. Bold values mean mediation effect value.***p < 0.001.

### Indirect Effects

This research conducted HLM to examine the mediating role of teamwork competence in the relationship between technical competence, transactive memory, and task performance. According to Baron and Kenny (1986), three conditions should be satisfied: (1) there is a significant relationship between the independent variables (i.e., technical competence and transactive memory) and the dependent variable (i.e., task performance); (2) the mediator (i.e., teamwork competence); and (3) After adding the mediator variable, the relationship between the independent variable and the dependent variable decreases or becomes insignificant.

Model 4 and 5 predict that teamwork competence will moderate the relationship between technical competence, transactive memory, and task performance. The results were consistent with our predictions. Compared with Model 1 and Model 3, after adding teamwork competence, the effects of technical competence (*γ*_10_ = 0.120, *p* > 0.05) and transactive memory (*γ*_01_ = 0.292, *p* > 0.05) on task performance were significantly reduced. This suggests that teamwork competence fully mediates the significant positive effects of technical competence (H1) and transactive memory (H3) on task performance, supporting Hypotheses 4 and 5. Given the limited sample size, the data were reanalyzed by MLwiN and 500 bootstrapping (Carpenter et al., 2003). The results are consistent, with coefficient variances below 0.05. This shows that our estimates are unbiased.

### *Post-hoc* Analysis

The first part of our *post-hoc* analysis focuses on the relationship between technology competence and teamwork competence (*γ*_10_ = 0.590, *p* < 0.001). Research indicated that there is a convergence of media technologies (Deuze, 2005; Williams and Tkach, 2021). As a result, reporters have to produce news content suitable for publication in various media formats through various channels and platforms (e.g., web, television, and social media). This could elevate the interdependence between individual reporters as they come to terms with multimedia technologies for producing and disseminating news content. In other words, teamwork competence plays an increasingly important role in actualizing the potential of digital technologies.

The second part of the *post-hoc* analysis addresses the relationship between transactive memory and teamwork competence (*γ*_01_ = 0.511^**^, *p* < 0.05). Indeed, the non-routine nature of journalism requires more and different sources and viewpoints. Transactive memory prepares reporters to bridge boundaries between their individual disciplines and specialties, facilitating collaboration with others.

## Discussion and Implications

The results confirmed the following positive direct and indirect relationships: (1) from technical competence to individual task performance; (2) from teamwork competence to individual task performance; (3) from transactive memory to individual task performance; (4) teamwork competence mediate technical competence to individual task performance; and (5) teamwork competence mediate transactive memory to individual task performance. Overall, given the full mediation role of teamwork competence, the bigger picture emerges that neither technical competence nor transactive memory necessarily translates directly into improved task performance for individual reporters. Instead, journalists must have the teamwork skills needed to translate technical competence and transactive memories into enhanced personal performance. Examining direct and indirect effects is important as it improves our understanding of the mechanisms by which mediating technical competence and transactive memory influence individual performance (teamwork competence in this study).

The second contribution of this research is corroboration that transactive memory improves the individual performance of knowledge workers. Past research has shown that transactive memory allows teams with limited human resources to collect and store knowledge and information, thereby improving team performance (Kim et al., 2021; Wang and Wu, 2021). This research responds to the research of which investigate the impact of transactive memory on individual performance. With the aid of HLM analyses, this research provides empirical confirmation of transactive memory on individual performance.

Prior studies indicate that the transformation of transactive memory into team performance is through the mechanism of interpersonal interaction that is facilitated by the team process (Faraj and Sproull, 2000). The results indicate that the effects of transactive memory on individual performance are fully mediated by the individuals’ teamwork competence. This means that given the same team process for leveraging transactive memory, individual team members’ teamwork competence can further explain distinct individual performance in a fast-paced team. Indeed, individual teamwork competence is a prerequisite for effective interpersonal interaction. People with high teamwork competence tend to be cooperative, trusting, and trustworthy, and show concern for others. This makes social interaction and exchange easier and safer (Feller et al., 2008). Thus, people can further induce active, voluntary, and productive participation from others, and ultimately leverage transactive memory to the benefit of individual performance (Bachrach et al., 2019).

Finally, the findings also suggest that teamwork competence mediates the relationship between technology competency and individual performance. Not surprisingly, digital technology offers individual journalists an easy and quick way to access information and spread their products. For example, celebrities or politicians with access to social media platforms, such as Facebook and Twitter, can provide and spread information fast. Individual news reporters thus can access the information and set the agenda of public issues through the dissemination of frequent and fast information sharing on social media.

However, this kind of journalism is not without ethical concerns over personal privacy, intellectual property, and fact-checking (Light and McGrath, 2010). Technology-driven journalism is increasingly drawing news reporters and news organizations into the relentless competition between commercial interests, sometimes at the expense of the professional value of journalism. With teamwork competence, news reporters, facilitated by digital technologies, are more likely to widen their reach and enhance their individual performance, while maintaining high standards of professional integrity.

Practically, our findings have several implications for curriculum design and execution for journalism-related education. This study suggests two basic fields of competence, namely, technology competence and teamwork competence, that could be incorporated into traditional journalism education which tends to be related to the humanities and the humanistic social sciences, such as political theory, philosophy, history, art, and literature (Donsbach, 2014). First, with the technological changes brought about by ICT, journalists must learn to work across different media technologies. However, instead of a simple focus on training in the latest technology, journalism students should learn to adapt to different technologies, because ICT is constantly changing. This means a deeper understanding of new technologies *per se*. Cross-disciplinary programs between journalism and other disciplines (e.g., engineering, information management) or the formation of interdisciplinary student teams (e.g., journalism and engineering students), could help journalism students to achieve a deeper understanding of new technologies (Angus and Doherty, 2015). Second, in the digitalized world, teamwork is becoming even more relevant for journalists due to the convergence of media technologies. Team-based learning should help develop teamwork competence in journalism students. Student teams should be routinely engaged in solving real-world problems across different domains. Therefore, the ability to understand problems outside their specialties, and the skills to collaboratively solve problems will be developed. Finally, teamwork competence is never easy to learn and measure. IT-based learning and assessment tools thus can be introduced (Fidalgo-Blanco et al., 2015). The tools will help measure individual contributions and provide timely feedback on the team process for preventing problems, carrying out corrective measures, and ultimately improving the learning process of teamwork.

## Conclusion and Limitations

The traditional journalism curriculum tends to emphasize the role of individualistic journalists who enjoy a high degree of autonomy and reputation in society. Our research shows that transactive memory and technology competency can help improve journalists’ job performance. More importantly, teamwork competence fully mediates interpersonal relationships. Our findings suggest two key areas of competence that journalists should master in the digitalized world, with teamwork competence playing the central role. In terms of the relationship between journalism and technology, journalism gatekeeping has moved from Web 1.0 to Web 2.0. News is not a completed factual report, but a process of engaging in dialogue with the audience (Robinson, 2011). Therefore, this study explores the impact of technological capabilities on the performance of news teams. How to properly integrate news technology and journalism value into higher education is another complex issue (Lin, 2021). From the competence point of view, this study also proposes the importance of team management. The media supervisors expect students, in addition to journalism, to have interpersonal communication, self-regulated learning, and problem-solving skills. These competencies could be developed from teamwork in school.

However, this study has some limitations. Common method variance (CMV) may overstate or understate the magnitude of relationships between variables because data are collected from a single source and perception-based measures are used. Therefore, this research examines Harman’s univariate test for all items. No single factor emerged, and no single factor explained most of the variance. Furthermore, it also applied confirmative factor analysis (CFA) by allowing items to load on their theoretical constructs, as well as on a latent common measurement factor (Podsakoff et al., 2003). Although this result shows that the structural patterns both with and without the latent measurement factor are significantly different (△*χ^2^* = 781.50, *p* < 0.00) and CMV is not a serious problem in our study, the further research could collect the data from a different time or different sources to reduce the bias.

Besides, our findings are based on the news industry in Taiwan, which is known for its hyper-competitiveness and fast pace, following a proliferation of over 180 TV stations that provide a constant stream of content. However, given the knowledge-intensive and fast-paced nature of news production, our findings may still be generalized to other types of tightly scheduled knowledge-based work, such as management consultancy or system development.

## Data Availability Statement

The raw data supporting the conclusions of this article will be made available by the authors, without undue reservation.

## Ethics Statement

The studies involving human participants were reviewed and approved by National Cheng Kung University Governance Framework for Human Research Ethics Case Number: 110–560. The ethics committee waived the requirement of written informed consent for participation.

## Author Contributions

C-HW builds up the theoretical background and provides the filed experiences. GH-WL collects the related literature and proposes hypotheses. C-DY designs the methodology and data analysis who is also the correspondent author. All authors cooperate to discuss the content of the manuscript, collaborate, and complete the final version of the manuscript.

## Funding

This research is sponsored by Ministry of Science and Technology, Taiwan, R.O.C. under Grant no. MOST 110-2410-H-019-017.

## Conflict of Interest

The authors declare that the research was conducted in the absence of any commercial or financial relationships that could be construed as a potential conflict of interest.

## Publisher’s Note

All claims expressed in this article are solely those of the authors and do not necessarily represent those of their affiliated organizations, or those of the publisher, the editors and the reviewers. Any product that may be evaluated in this article, or claim that may be made by its manufacturer, is not guaranteed or endorsed by the publisher.
